# Retinoic Acid Improves Morphology of Cultured Peritoneal Mesothelial Cells from Patients Undergoing Dialysis

**DOI:** 10.1371/journal.pone.0079678

**Published:** 2013-11-04

**Authors:** Carmen Retana, Elsa I. Sanchez, Sirenia Gonzalez, Alejandro Perez-Lopez, Armando Cruz, Jesus Lagunas-Munoz, Carmen Alfaro-Cruz, Socorro Vital-Flores, José L. Reyes

**Affiliations:** 1 Pharmacology Department Centre for Research and Advanced Studies National Polytechnic Institute, Mexico, D.F., Mexico; 2 Physiology, Biophysics and Neurosciences Department Centre for Research and Advanced Studies National Polytechnic Institute, Mexico, D.F., Mexico; 3 Central Laboratories Centre for Research and Advanced Studies National Polytechnic Institute, Mexico, D.F., Mexico; 4 Nephrology Department, Hospital Central Norte de Petroleos Mexicanos, Mexico, D.F., Mexico; 5 Nephrology and Gynecology Departments Unidad Medica Alta Especialidad, Hospital General la Raza, Instituto Mexicano del Seguro Social, Mexico, D.F., Mexico; 6 Nephrology. Dept. Hospital Regional No. 1 del Instituto Mexicano del Seguro Social, Mexico, D.F., Mexico; 7 Department of Nephrology, Hospital Juarez de Mexico, Mexico, D.F., Mexico; University of Louisville, United States of America

## Abstract

Patients undergoing continuous ambulatory peritoneal dialysis are classified according to their peritoneal permeability as low transporter (low solute permeability) or High transporter (high solute permeability). Factors that determine the differences in permeability between them have not been fully disclosed. We investigated morphological features of cultured human peritoneal mesothelial cells from low or high transporter patients and its response to All trans retinoic Acid (ATRA, vitamin A active metabolite), as compared to non-uremic human peritoneal mesothelial cells. Control cells were isolated from human omentum. High or low transporter cells were obtained from dialysis effluents. Cells were cultured in media containing ATRA (0, 50, 100 or 200 nM). We studied length and distribution of microvilli and cilia (scanning electron microscopy), epithelial (cytokeratin, claudin-1, ZO-1 and occludin) and mesenchymal (vimentin and α-smooth muscle actin) transition markers by immunofluorescence and Western blot, and transforming growth factor β1 expression by Western blot. Low and high transporter exhibited hypertrophic cells, reduction in claudin-1, occludin and ZO-1 expression, cytokeratin and vimentin disorganization and positive α-smooth muscle actin label. Vimentin, α-smooth muscle actin and transforming growth factor- β1 were overexpressed in low transporter. Ciliated cells were diminished in low and high transporters. Microvilli number and length were severely reduced in high transporter. ATRA reduced hypertrophic cells number in low transporter. It also improved cytokeratin and vimentin organization, decreased vimentin and α-smooth muscle actin expression, and increased claudin 1, occludin and ZO-1 expression, in low and high transporter. In low transporter, ATRA reduced transforming growth factor-β1 expression. ATRA augmented percentage of ciliated cells in low and high transporter. It also augmented cilia length in high transporter. Alterations in structure, epithelial mesenchymal markers and transforming growth factor-β1expression were differential between low and high transporter. Beneficial effects of ATRA were improved human peritoneal mesothelial cells morphology tending to normalize structures.

## Introduction

In continuous ambulatory peritoneal dialysis (CAPD) peritoneum constitutes the permeability membrane across which ultrafiltration and diffusion occur. Patients are classified according to their peritoneal transport as: high or “fast” transporters, high-average, low-average and low or “slow” transporters. High transporters (HT) display a rapid transport of uremic toxins and solutes from the bloodstream to the dialysate. Fast transport rate causes rapid glucose absorption and loss of the osmotic gradient, leading to lower ultrafiltration [1]. Low transporters (LT) depict low glucose absorption, therefore they maintain osmotic gradient for a longer time, which makes ultrafiltration more efficient [2]. 

Peritoneum is lined by a monolayer of mesothelial cells. Mesothelium participates in fluid and solute transport during CAPD. Morphological and structural features of human peritoneal mesothelial cells (HPMCs) from LT or HT are ill defined. Mesothelial cells possess features of epithelial cells with a polygonal, cobblestone appearance. They have specialized molecules for transport of water and solutes, and rest upon a basement membrane [3,4]. Abundant microvilli and occasional cilia are found on their luminal surface. Microvilli increase peritoneal surface area for transport of solutes and protect mesotelium from frictional injury by entrapment of water and secretion of serous exudates, whereas cilia regulate the secretion of surfactants [5]. They enable cells to sense and respond to their microenvironment [6,7]. A reduction in the number of these structures on mesothelial cells would therefore have an untoward effect on peritoneal function and transport.

CAPD induces deleterious changes in mesothelial cells, such as loss of microvilli, widening of the intercellular spaces, and exfoliation [8,9]. After exposure to nonphysiological dialysis solutions, mesothelial cells undergo epithelial to mesenchymal transition (EMT) [10,11]. During EMT, they show a progressive loss of epithelial phenotype and acquire a fibroblast-like phenotype with loss of their permeability characteristics [12,13]. In addition, mesothelial cells gradually lose their typical cytoskeleton organization and epithelial cell markers (E-cadherin and cytokeratins), and progressively upregulate expression of mesenchymal markers (vimentin and α-smooth muscle actin (α-SMA)) [14,15]. Transforming growth factor β1( TGF-β1) is a key mediator of EMT in several cells [15,16], including cultured HPMCs [17]. 

Retinoids are important regulators of epithelial differentiation and proliferation. Induction of differentiation by retinoic acid has been observed in various cell systems [18,19]. Retinoids are potent regulators of epithelial morphology in HPMCs [20]. 

The aim of this study was to compare morphological and structural characteristics (cilia and microvilli) as well as markers of EMT in cultured HPMCs from CAPD patients with LT or HT behaviour, and their response to all trans retinoic acid (ATRA). 

## Subjects and Methods

### Ethics Statements

This research was carried out in accordance with Good Clinical Practice guidelines, applicable regulations as well as the ethical principles originated in the Declaration of Helsinki. The present study was approved by the Ethics Committee of “Hospital de Gineco-obstetricia No. 3. Centro Medico Nacional La Raza. Instituto Mexicano del Seguro Social” (Mexico). Written informed consent was obtained from omentum donors. Verbal consent for peritoneal dialysis bags was agreed with the patient after an explanation given by the nephrologist of the purpose of the study. Since this material is usually discarded, there were no objections from the Ethics Committee.

### Patients

Peritoneal biopsies were obtained from 12 non-uremic female patients (38.4 ± 2.0 years, range 23 to 48 years), undergoing abdominal surgery, after informed consent. They integrate the control group. Effluent-derived mesothelial cells were obtained from 24 CAPD patients, including 15 low transporters and 9 high transporters receiving glucose-based dialysis solutions at 1.5 or 2.5 %. Clinical characteristics are shown in Table 1. Diabetic patients were not included.

**Table 1:** Clinical features of high and low transporters.

	**Low transporters**	**High transporters**
	**N=15**	**N=9**
AGE	27.9 ± 2.0 (13-79)	36.3 ± 8.1 (13-71)
MALE/FEMALE	5/10	6/3
POLYCYSTIC KIDNEY	2	0
ARTERIAL HYPERTENSION	3	1
GLOMERULONEPHRITIS	1	0
NEPHROANGIOSCLEROSIS	0	2
UNKNOWN ETIOLOGY	9	6
DIALYSIS DURATION (MONTHS)	29.6 ± 7.0 (1-84)	17.0 ± 6.6 (1-48)
D/P CREATININE 4H	0.5 ± 0.03	0.8 ± 0.03 *
GLUCOSE (mg/dL)	90.2 ± 2.1	88.6 ± 3.3
ALBUMIN (g/dL)	3.68 ± 0.09	3.64 ± 0.09
BUN (mg/dL)	67.8 ± 5.6	56.8 ± 3.7
SERUM UREA (mg/dL)	144.1 ± 12.0	124.7 ± 9.5
SERUM CREATININE (mg/dL)	8.6 ± 0.6	9.2 ± 0.7
PERITONITIS EPISODES (6 MONTHS PREVIOUS TO STUDY)	7	3

Abbreviations: BUN, blood urea nitrogen; D/P creatinine, dialysate/plasma creatinine. Mean ± standard error of the mean.*p<0.*05* low transporters versus high transporters.

### Antibodies and chemicals

human TGF-β1 (Cat. No T 0438) and ATRA (Cat.no R-2625) were from Sigma Aldrich (Saint Louis, MO); mouse anti-acetylated α- tubulin (Cat. no 32-2700), mouse anti-cytokeratin (Cat. no 18-0213), rabbit anti-claudin-1 (Cat. no 51-9000), rabbit anti-occludin (Cat. no 71-1500), rabbit anti-ZO-1 (Cat. no 61-7300) and mouse anti- vimentin (Cat. no 18-0052) were obtained from Zymed (South San Francisco, CA); α-SMA (Cat. no. BCL171) was from Merck Millipore (CA), mouse anti-actin was kindly donated by Dr. Jose Manuel Hernandez (Department of Cell Biology, Centre for Research and Advanced Studies National Polytechnic Institute, Mexico, d.f., Mexico) [21]. Secondary antibodies: Alexa fluo 594 rabbit anti-mouse (Cat. no A-11062), Alexa fluo 488 goat anti-mouse (Cat. no A-11001) and ToPro-3 iodide (Cat. no T360) were from Invitrogen, and HRP anti-mouse was from Pierce Biotechnology Inc. USA.

### Cell isolation

Biopsies were collected in cold PBS and incubated with 0.25% trypsin and 0.1% EDTA for 30 min. Mesothelial cells were separated by centrifugation and cultured in Dulbecco´s Modified Eagle´s Medium: nutrient mixture F-12 (Ham) (1:1), supplemented with 12% vol/vol fetal bovine serum, penicillin (100 U/mL), streptomycin (100 µg/mL), L-glutamine (2 μg/mL), transferrin (5 μg/mL), insulin (5 μg/mL) and hydrocortisone (0.4 μg/mL). Once cells were in passage 3, they were grown until confluence with medium containing ATRA (50, 100 or 200 nM). Medium was replaced every two days.

Isolation of effluent-derived mesothelial cell was performed according to Diaz et al [22]. Briefly, peritoneal dialysates collected overnight were drained and processed immediately. Dialysis bag content was centrifuged for 20 min, at 327 g, at 3 °C. Pellet was resuspended, platted and maintained in culture media, replaced every two days. Culture media were as described for control cultures. Once cells were in passage 3, they were cultured until confluence with medium containing ATRA (50, 100 or 200 nM). Medium was replaced every two days.

### Scanning electron microscopy

Cells were seeded on collagen I-covered glass coverslips (modified method of Bornstein M.B) [23] and cultured in medium with ATRA 0, 50, 100 and 200 nM until confluence. Then, they were fixed with 2.5% glutaraldehyde for 1h at 37 °C. Monolayers were post-fixed with osmium tetroxide for 1 h. Cells were dehydrated in an ethanol ascending series (from 50% to 100%), critical-point dried using a Sandri-780A apparatus (Tousimis), gold-coated with a Desk II Gold sputter-etch unit (Denton Vacuum Inc.), and examined with a Jeol JSM-6510LV scanning electron microscopy that was operated at 25 kV with a working distance of 9-11 mm.

### SEM image evaluation

Images from 10 cells from at least 3 cultures obtained from three different patients were randomly captured to measure cilia length. Two fields from each cell were captured (x10000), to quantify microvilli number and length. In HT, microvilli tend to fuse instead of being single structures as in control and LT. For counting microvilli in HT, each group of fused microvilli was counted as one. Microvilli number and length, and cilia length were counted and measured directly from micrographs as described by Bird [24]. In addition, images of two fields were randomly captured (x300) in each condition to quantify the number of total, hypertrophic and flattened cells per field. Count was performed directly from micrographs.

### Immunofluorescence

Cells were cultured in the presence of ATRA 0, 50 and 100 nM. Monolayers were washed with PBS, fixed with cold methanol for 10 min at 4°C, rehydrated in PBS, permeabilized with 0.25% Triton X100 for 15 min, and then blocked with 0.5% BSA. They were incubated overnight with the primary antibody at 4 °C, followed by washes with PBS, and incubation with the secondary antibody for 1 h, at room temperature. Specimens were examined under confocal laser scanning microscope (Leica DMIRE-2, Germany).

### Percentage of ciliated cells

Cells were processed for immunofluorescence co-staining with anti-acetylated α-tubulin (a cilia component) and anti-nucleus (ToPro-3 iodide) antibodies. Total number of cells and number of ciliated cells were counted in duplicate from at least 3 experiments for each condition.

### Western blot

Cells were cultured in 100 mm x 20 mm dishes (Corning, NY, USA) with ATRA 0, 50 and 100nM. Monolayers were washed twice with cold PBS. Total fraction was extracted by adding (100μL) RIPA, PMSF (Sigma, Saint Louis, MO) and Complete (Roche Diagnostics, Mannhein, Germany) proteases inhibitors. Proteins were quantified by Lowry method (Bio Rad laboratories, CA), denatured by boiling and diluted 1:5 in Laemmli (Bio Rad laboratories, CA), urea buffer (0.5 M) and 2-mercaptoethanol (Bio Rad laboratories, CA). Samples were loaded on SDS-PAGE 10% gels and transferred to PVDF sheets (Amersham Biosci, UK). Nonspecific binding was blocked with 5% non-fat dry milk in TBS 1X containing 0.4% Tween 20, for 1 h, at room temperature. Bands were detected with specific antibodies against anti-cytokeratin (dilution 1:500), anti-vimentin (dilution 1:500), anti-α-SMA (dilution 1:500), anti-claudin-1 (dilution 1:1000), anti-ZO-1 (dilution 1:1000), anti-occludin (dilution1:1000) and chemiluminiscence (ECL plus Western blotting detections agents), detected in an EC3 imaging System (UVP, Biolmaging Systems, Cambridge, UK). Protein band density was quantified by transmittance densitometry (UVP Biolmaging Systems software, Cambridge, UK).

### Statistical analyses

Results are expressed as mean ± standard error of the mean (SEM). Data were examined using one way ANOVA with Tukey post test. P<0.05 was considered as significant. 

## Results

### Retinoic acid improved morphology and decreased number of hypertrophic cells in LT cultures

**Figure 1:**
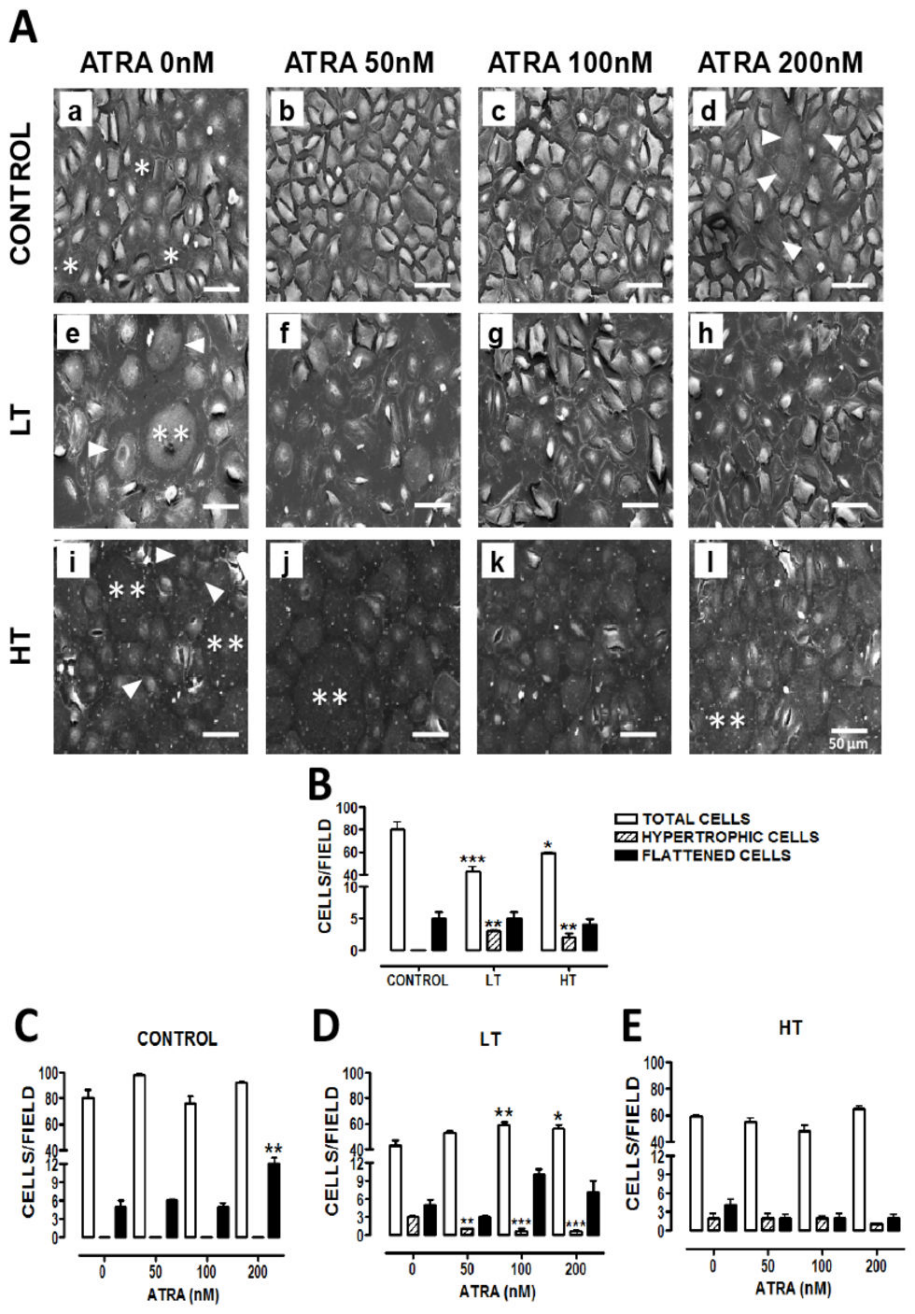
Retinoic acid improved cell morphology and decreased the number of hypertrophic cells in LT cultures. (**A**) Omentum-derived mesothelial cells (control) and effluent-derived mesothelial cells from LT and HT grown until confluence in the presence of ATRA (0, 50, 100 and 200 nM). LT cells exhibited an increase in their average size (e, arrowheads) and hypertrophy (e, double asterisk). In HT cultures epithelial (i, arrowheads) and hypertrophic (i, double asterisk) cells were both observed. ATRA reduced the presence of hypertrophic cells in LT, in a concentration-dependent manner (f, g and h). (B to E) Quantification of total (empty bars), hypertrophic (hatched bars) and flattened (filled bars) in mesothelial cells treated as in A. LT and HT exhibited less cells by field and more hypertrophic cells than control cultures (**B**). In LT cultures, ATRA decreased the number of hypertrophic cells (**D**). Scanning electron microscopy, x300, bar= 50µm. Representative photomicrographs were obtained by duplicate from three different patients. Mean ± SEM are shown. (**B**) ***P< 0.001 and *P<0.05 versus control total cells;**P<0.01 versus control hypertrophic cells. (**C**)**P<0.01 versus control flattened cells with ATRA 0 nM (**D**) **P<0.01 and *P<0.05 versus LT with ATRA 0nM; **P<0.01 and ***P<0.001 versus LT with ATRA 0 nM. ATRA, all trans retinoic acid; LT, low transporter; HT, high transporter.

Control cultured HPMCs showed polygonal cells (Figure 1A, panel a), with occasional flattened appearance (asterisks), suggesting cells in a proliferative phase. In the presence of ATRA, control cells (Figure 1A, panels b, c and d) showed cobblestone morphology and several flattened cells were observed at 200 nM (panel d, arrowheads). LT cells (Figure 1A, panel e) exhibited increased average cell size (arrowheads) compared to control, and hypertrophic cells (double asterisk). In LT, ATRA reduced cell size similar to control cultures and decreased the presence of hypertrophic cells in a concentration-dependent manner (Figure 1A, panels f, g and h). In HT (Figure 1A, panel i), epithelial (arrowheads) and hypertrophic cells (double asterisk) were both observed. ATRA did not have effect in these cultures. We quantified number of total cells. Compared with control cultures (80 ± 7) both LT (43 ± 4.3) and HT (59 ± 1.3) had less cells by field, being more evident in LT (Figure 1B). In control group, ATRA (200 nM) increased number of flattened cells (Figure 1C). In LT, it decreased the number of hypertrophic cells (Figure 1D). In HT, ATRA did not have effect (Figure 1E).

### ATRA improved cytokeratin organization and distribution in HPMCs from LT and HT

**Figure 2:**
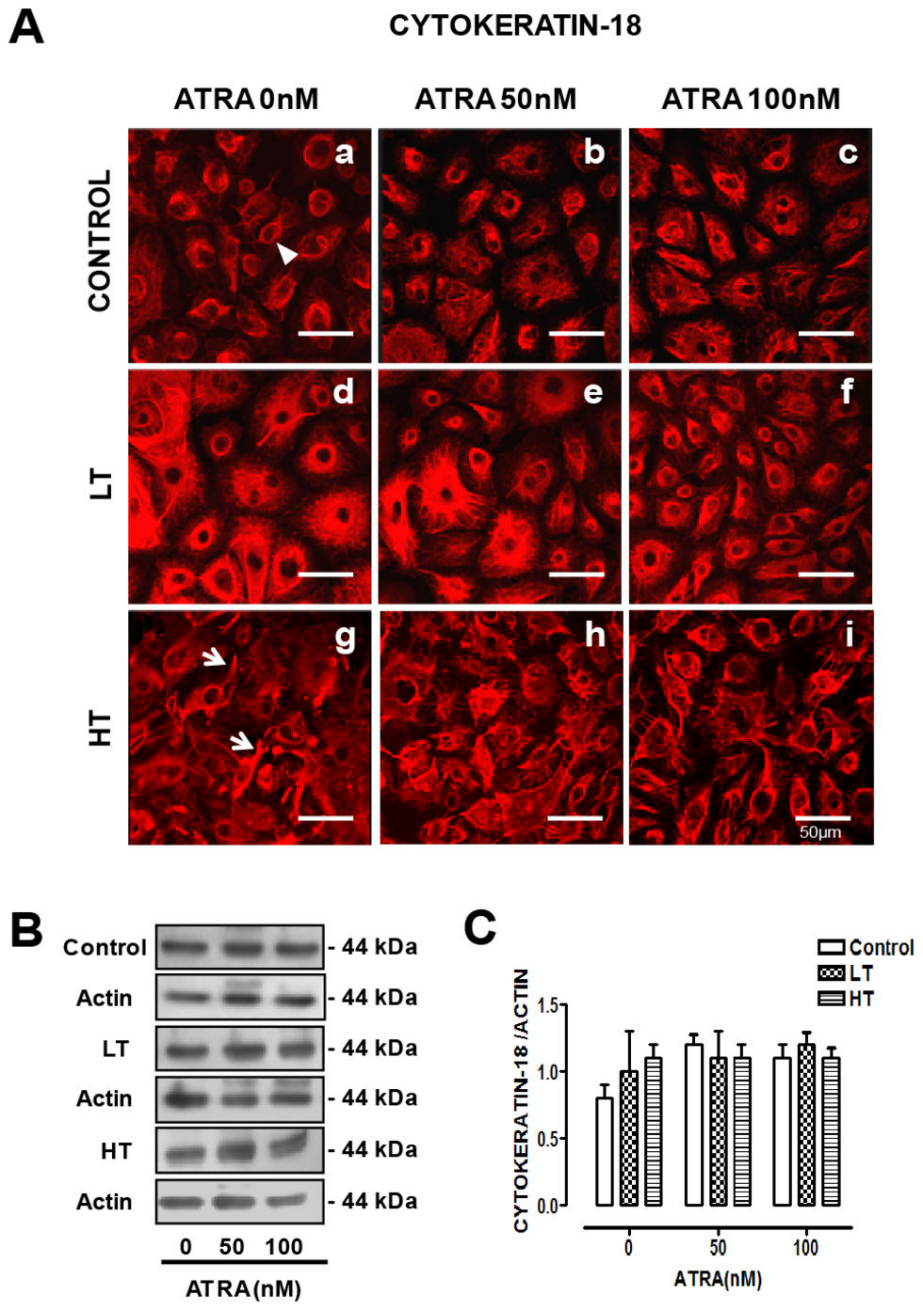
ATRA enhanced the organization of cytokeratin-18 in LT and HT cultures. (**A**) Immunofluorescence of omentum-derived mesothelial cells (control) and effluent-derived mesothelial cells from LT and HT grown until confluence in the presence of ATRA (0, 50 and 100 nM). Cells were labeled with anti-cytokeratin-18 (red). Cytokeratin-18 organization was altered in LT (d) and HT (g) cells. ATRA treatment reversed the cytokeratin-18 alterations observed in LT and HT cultures (f and i). (**B**) Western blot analyses and densitometry (**C**) showing the expression of cytokeratin-18 in total cell lysates of mesothelial cells treated as in A. ATRA did not modify cytokeratin-18 expression. Mean ± SEM of three individual experiments from three different patients are shown. ATRA, all trans retinoic acid; LT, low transporter; HT, high transporter.

Cytokeratin is a marker of epithelial phenotype. In control HPMCs, cytokeratin label was perinuclear (Figure 2A, panel a, arrowhead). In LT cells, perinuclear label was prominent and extended through the entire cellular body (panel d). In HT, cytokeratin was disorganized and some projections were observed (panel g, arrows). ATRA (100 nM) improved cytokeratin organization and distribution in LT (panel f) and HT (panel i). In control cultures, ATRA did not modify the pattern described (panels b and c). Western blot did not show differences in cytokeratin content among groups. ATRA did not have effect on cytokeratin expression (Figure 2, B and C). In addition to cytokeratin, expression of claudin 1, occludin and ZO-1 as epithelial markers were explored. Under basal conditions, LT and HT showed a significant reduction in the expression of claudin 1, occludin and ZO-1 compared to control (Table 2). ATRA treatment (50 and 100 nM), increased the expression of ZO-1 in LT and the expression of claudin-1, occludin and ZO-1 in HT (Table 2)

**Table 2:** All trans retinoic acid increased expression of epithelial markers (claudin 1, occludin and ZO-1).

	CLAUDIN 1 (DU)*	**OCCLUDIN**	**ZO-1**
**CONTROL**	**851 ± 4**	**294 ± 3**	**183 ± 0.8**
**LT**	**250 ± 3^A^**	**144 ± 2^E^**	**59 ± 0.4^I^**
**LT+ATRA 50 Nm**	**223 ± 13**	**104 ± 14**	**90 ± 7^K^**
**LT+ATRA 100 nM**	**179 ± 40**	**159 ± 12**	**113 ± 11^L^**
**HT**	**595 ± 0.4^B^**	**175 ± 2^F^**	**123 ± 0.8^J^**
**HT+ATRA 50 nM**	**973 ± 119^C^**	**340 ± 28^G^**	**389 ± 86^M^**
**HT+ATRA 100 nM**	**1014 ± 77^D^**	**366 ± 38^H^**	**247 ± 41^N^**

Epithelial markers quantification by Western blot. Human peritoneal mesothelial cells from patients with peritoneal low (LT) or high transport (HT) were grown until confluence in the presence of all trans retinoic acid (ATRA) 0, 50 or 100 nM. Mean± SEM of three experiments from three different patients. * DU= Densitometric units.

^A^ ^and B^ p<0.01 vs control claudin 1; ^C and D^ p<0.01 vs HT claudin 1.

^E^ ^and F^ p<0.01 vs control occludin; ^G and H^ p<0.01 vs HT occludin.

^I^ ^and J^ p<0.01 vs control ZO-1; ^K^ p<0.05 vs LT ZO-1; ^L^ p<0.01 vs LT ZO-1; ^M and N^ p<0.01 vs HT ZO-1.

### ATRA improved vimentin organization and reduced its expression in LT and HT

**Figure 3:**
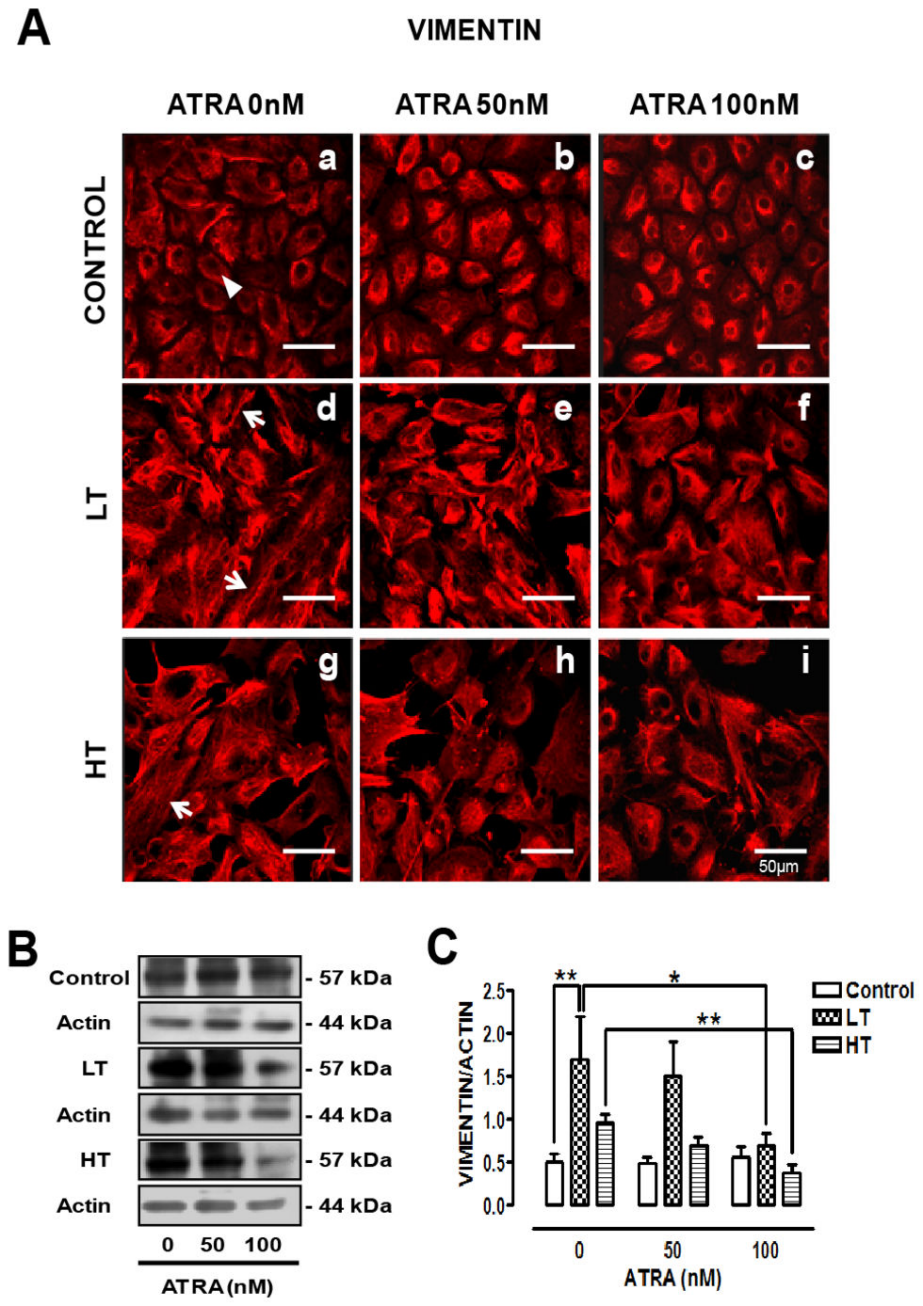
ATRA improved vimentin organization and decreased its expression. (**A**) Immunofluorescence of omentum-derived mesothelial cells (control) and effluent-derived mesothelial cells from LT and HT grown until confluence in the presence of ATRA (0, 50 and 100 nM). Cells were labeled with anti-vimentin (red). In LT cultures (d), vimentin immunofluorescence was more intense and exhibited elongated fibers (arrows). These fibers were also observed in HT cultures (g, arrows). ATRA improved vimentin organization in LT and HT, being more notable in LT (f). (**B** and **C**) Western blot analyses for vimentin in total cell lysates of mesothelial cells treated as in A. Vimentin expression was augmented in LT and HT cultures. ATRA reduced the vimentin content in LT and HT. Mean ± SEM of three individual experiments from three different patients are shown. **P<0.01 versus control; *P<0.05 versus LT with ATRA 100 nM and **P<0.01 versus HT with ATRA 100 nM. ATRA, all trans retinoic acid; LT, low transporter; HT, high transporter.

Vimentin showed perinuclear and cytosolic location in control HPMCs (Figure 3A, panel a, arrowhead). In LT, vimentin label was intense with a fibrillar pattern (panel d, arrows). This pattern was also observed in HT (panel g, arrow). In LT (panel f) and HT (panel i), ATRA (100 nM) improved vimentin distribution. In control culture there was no change. Western blot showed increased vimentin expression in LT and HT, more evident in LT. ATRA (100 nM) decreased vimentin expression in these cultures, without change in control (Figure 3, B and C).

### ATRA reduced α-SMA expression in LT and HT

**Figure 4:**
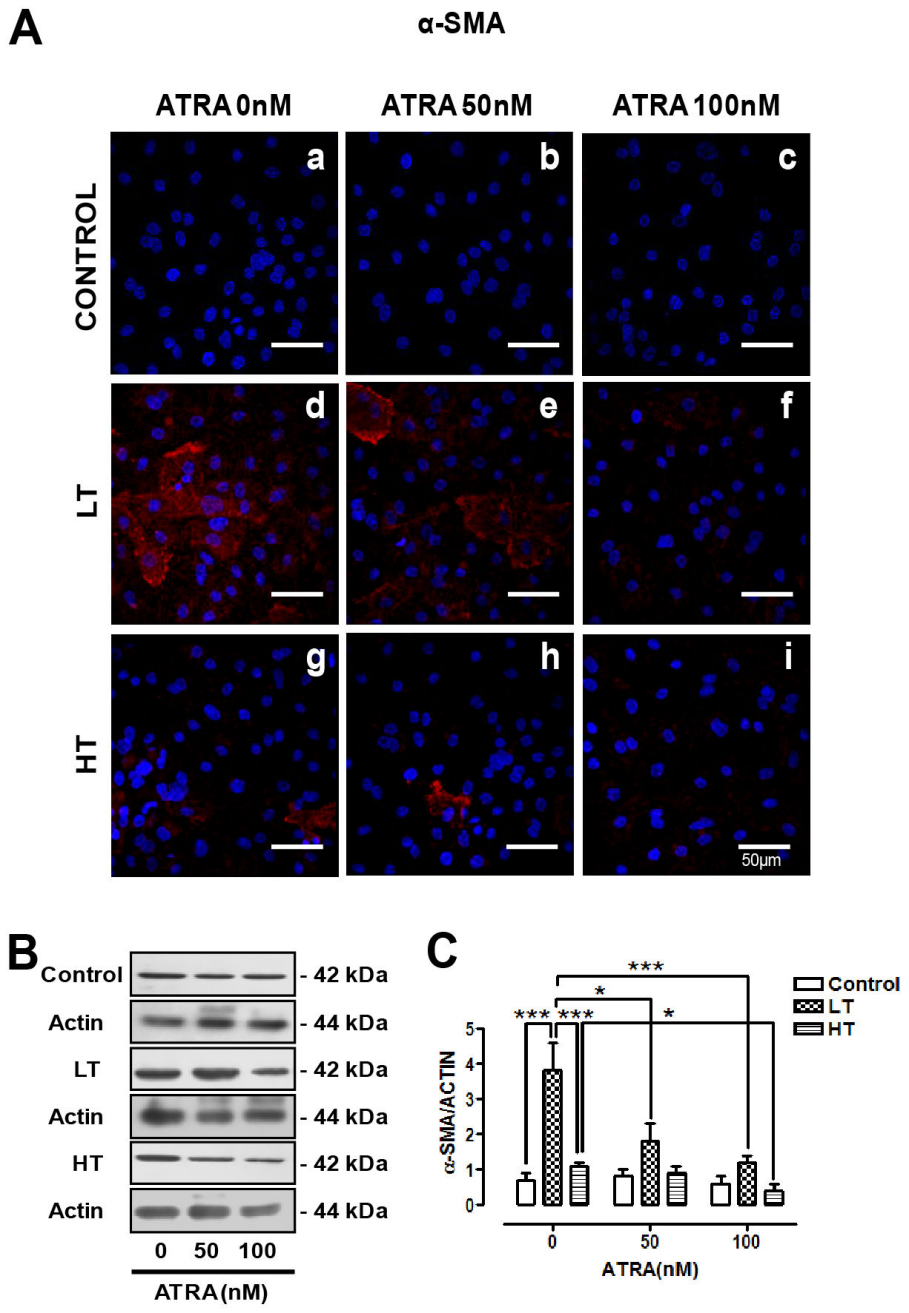
ATRA decreased α-SMA expression in LT and HT cultures. (**A**) Immunofluorescence of omentum-derived mesothelial cells (control) and effluent-derived mesothelial cells from LT and HT grown until confluence in the presence of ATRA (0, 50 and 100 nM). Cells were labeled with anti-α-SMA (red). Nuclei were labeled with ToPro-3 (blue). LT and HT cultures were positive for α-SMA staining (d and g). (**B** and **C**) Western blot analyses for α-SMA in total cell lysates of mesothelial cells treated as in A. α-SMA expression was importantly augmented in LT cultures. ATRA decreased α-SMA content in LT and in HT cultures. Mean ± SEM of three individual experiments from three different patients are shown. (**C**) ***P<0.001 versus control; ***P<0.001 versus HT; *P<0.05 and ***P<0.001 versus LT with ATRA 0 nM; *P<0.05 versus HT with ATRA 0 nM. ATRA, all trans retinoic acid; α-SMA, α-smooth muscle actin; LT, low transporter; HT, high transporter.

In control cultures, α-SMA label was absent (Figure 4A, panel a). In contrast, LT (panel d) and HT (panel g) were positive for α-SMA, being more evident in LT. ATRA (50 or 100 nM), decreased α-SMA in LT (panels e and f) and HT (panels h and i) in a concentration-dependent manner. Western blot showed that α-SMA was significantly augmented in LT compared with control and HT cultures (Figure 4C). ATRA decreased α-SMA expression in LT and HT (Figure 4, B and C), without change in control. 

### TGF-β1 overexpression in LT cultures was reversed by ATRA

**Figure 5:**
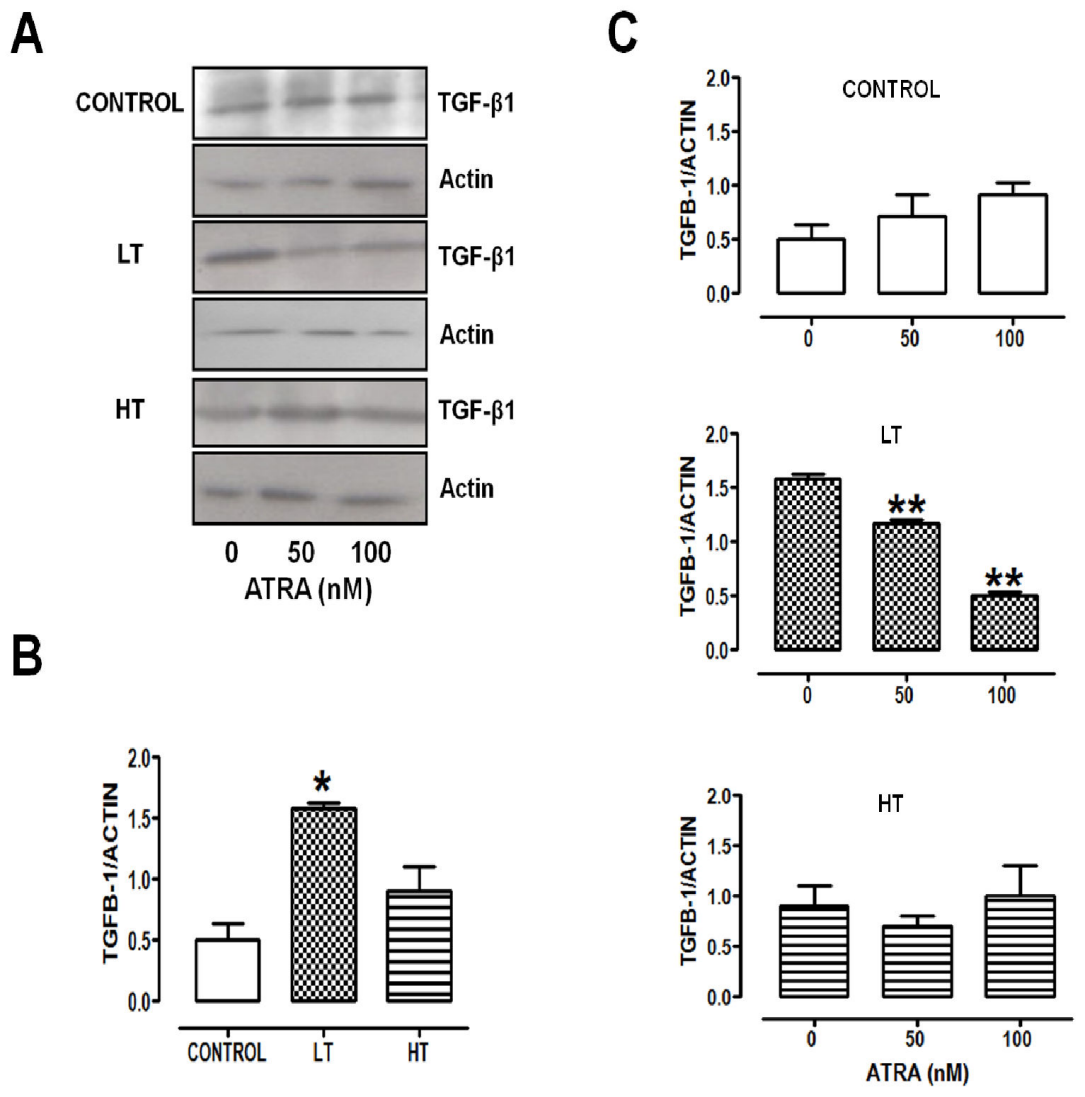
ATRA decreased TGF-β1 content in LT cultures in a concentration-dependent manner. (A to C) Western blot analysis and densitometry of TGF-β1in total lysates of omentum-derived mesothelial cells (control) and effluent-derived mesothelial cells from LT and HT grown until confluence in the presence of ATRA (0, 50 and 100 nM). TGF-β1 expression was increased in LT cultures (**B**). ATRA reduced the expression of TGF-β1 in these cultures (**C**). Media ± SEM of three individual experiments from three different patients are shown. (**B**) *P<0.05 versus control. (**C**) **P<0.01 versus LT with ATRA 0 nM. ATRA, all trans retinoic acid; TGF-β1 , Transforming growth factor β1; LT, low transporter; HT, high transporter.

HPMCs exposure to high glucose or peritoneal dialysis fluid enhances TGF-β1 expression [25]. Under basal conditions, LT cultures expressed more TGF-β1 compared with control and HT (Figure 5, A and B). ATRA (50 or 100 nM) decreased the expression of this profibrotic factor in a concentration-dependent manner only in LT (Figure 5, C). 

### HT cilia exhibited altered morphology

**Figure 6:**
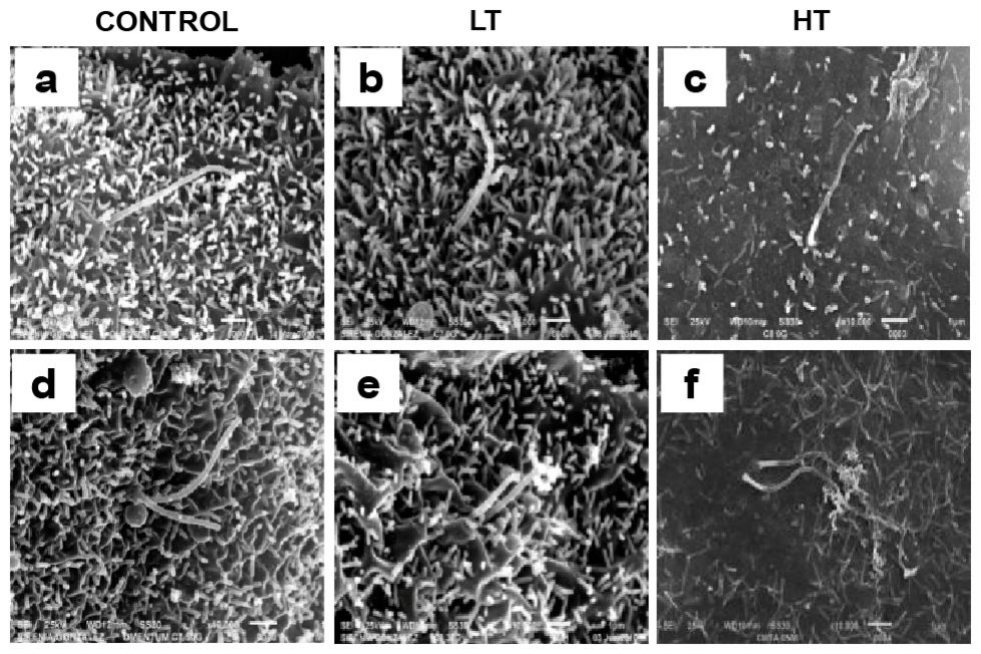
Cilium morphology in peritoneal mesothelial cells from LT and HT. Cells with one or two cilia were observed in omentum-derived mesothelial cells (a and d) and effluent-derived mesothelial cells from LT (b and e) and HT (c and f). Cilia in control (a) and LT (b) displayed a regular and continuous profile, while cilia in HT cells showed thinning from the base to the tip (c, arrow). Scanning electron microscopy, x10000, bar=1µm. Representative photomicrographs of three individual experiments from three different patients are shown. HPMCs, human peritoneal mesothelial cells; LT, low transporter; HT, high transporter.

In control, LT and HT cultures, cells with one (Figure 6, panels a, b and c) or two (Figure 6, panels d, e and f) cilia were observed. Cilia in control (Figure 6, panel a) and LT (Figure 6, panel b) displayed a regular and continuous profile, while cilia in HT cells showed thinning from the base to the tip (Figure S1, panels a and b, arrows). It was noteworthy that cilia morphology was more diverse in HT, than in control or LT, displaying cells with three cilia (Figure S1, panels d and e, arrowheads). This characteristic was observed in 6.6% of HT cells, but not in control or LT. In HT, cilia seem to be fused at their tips (Figure S1, panels c, d and e) and also showed an irregular pattern, with a “pearl necklace” (Figure S1, panels c and d, empty arrowhead) or “branched” (panel f, empty arrowhead) appearance. In several HT cells, the ciliary tip was laying on the cell surface (Figure S1, panels g and h, white arrows). 

### ATRA increased cilia length and number of ciliated cells in HPMCs

**Figure 7:**
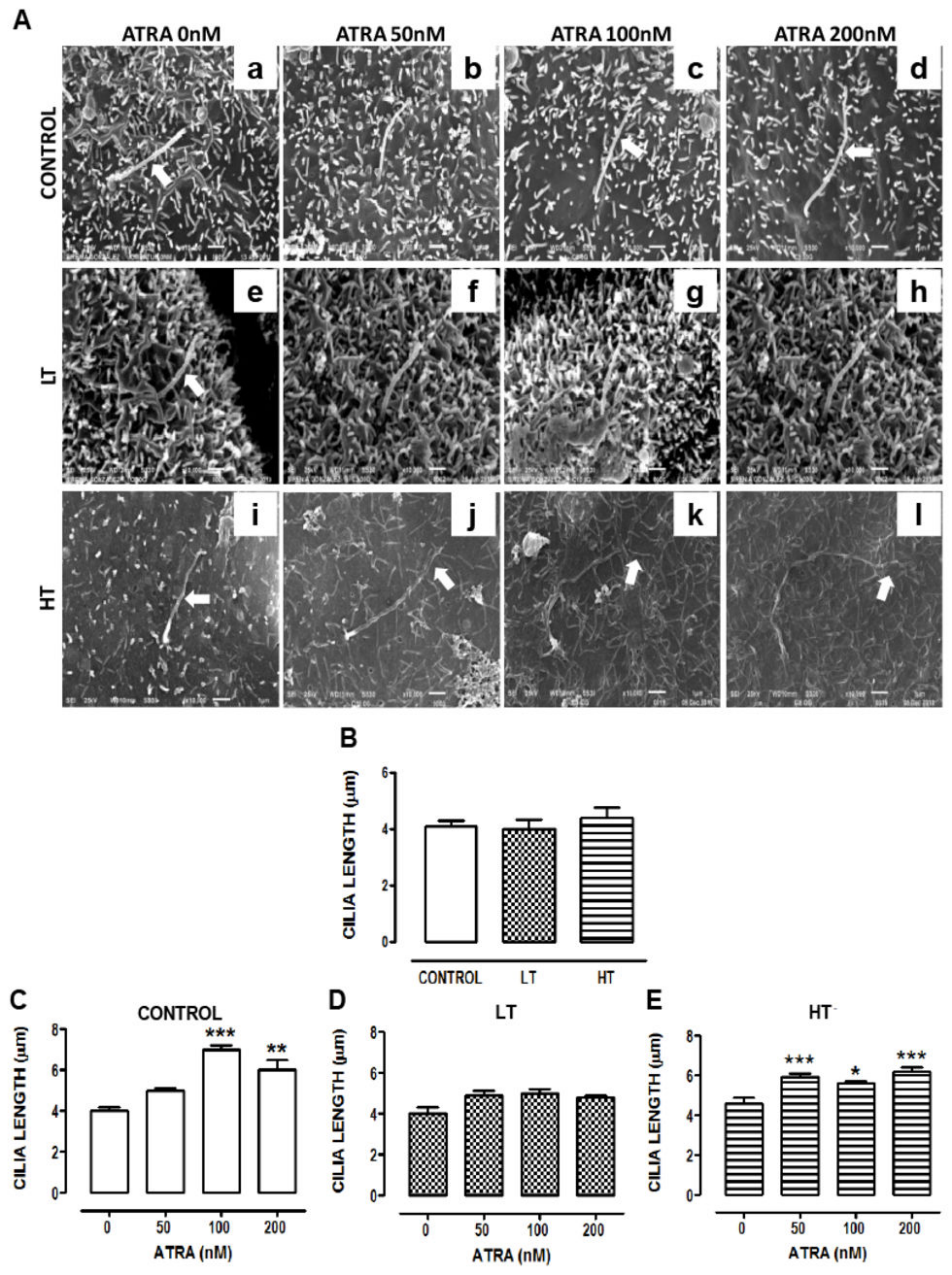
Retinoic acid increased the cilia length in HT. (**A**) Omentum-derived mesothelial cells (control) and effluent-derived mesothelial cells from LT and HT grown until confluence in the presence of ATRA (0, 50, 100 and 200 nM). ATRA increased the cilia length in control (c and d, white arrows) and HT (j, k and l, white arrows) cultures, whereas in LT it did not have effect (B to E) Measurement of cilia length in mesothelial cells treated as in A. Scanning electron microscopy, x10000, bar=1µm. Representative photomicrographs of three individual experiments from three different patients. Mean ± SEM. (**C**) ***P<0.001 and **P<0.01 versus control with ATRA 0 nM. (**E**) ***P<0.001 and *P<0.05 versus HT with ATRA 0 nM. ATRA, all trans retinoic acid; LT, low transporter; HT, high transporter.

**Figure 8:**
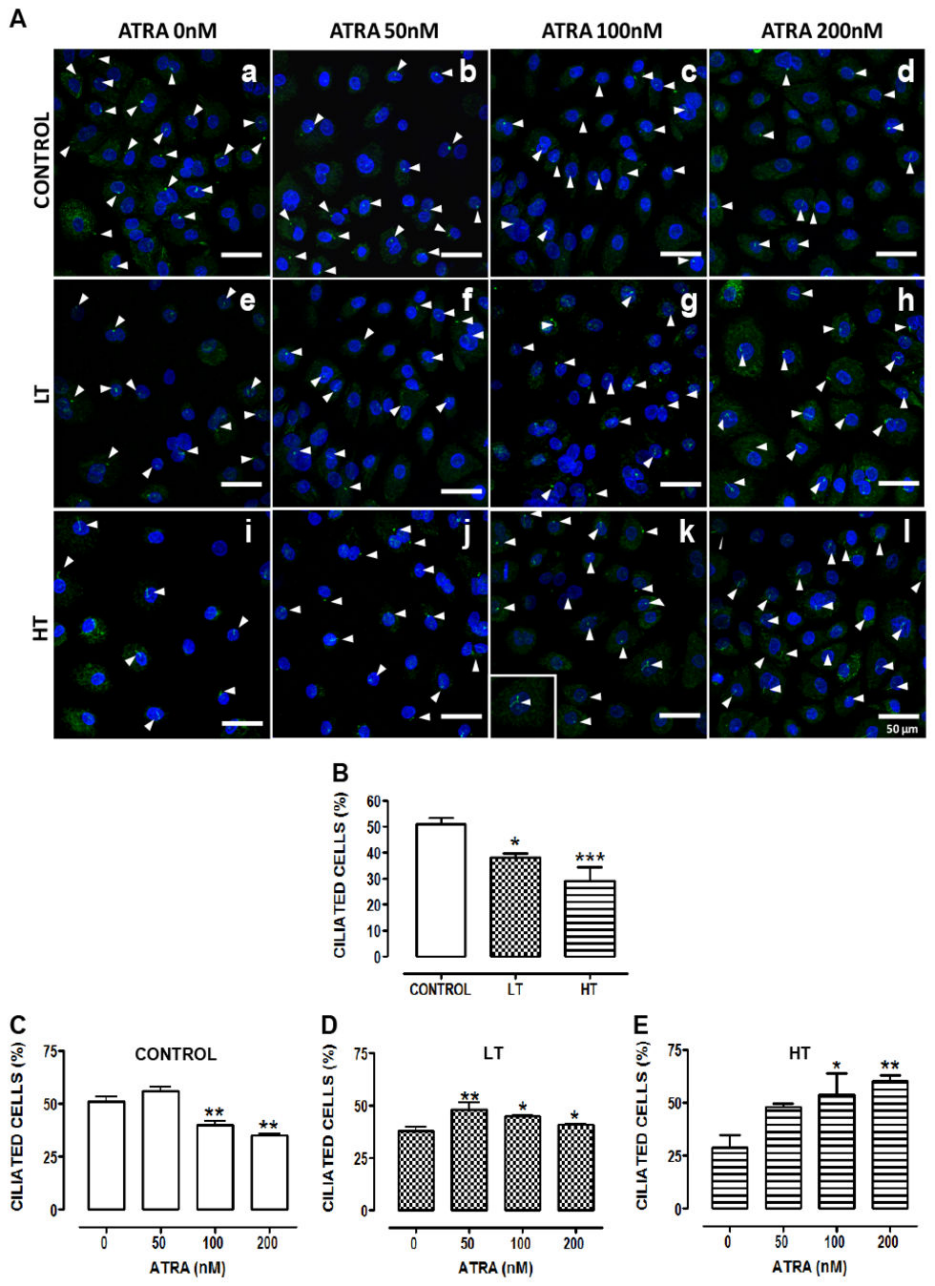
ATRA increased the number of ciliated cells in HT and LT. (**A**) Immunofluorescence of omentum-derived mesothelial cells (control) and effluent-derived mesothelial cells from LT and HT grown until confluence in the presence of ATRA (0, 50, 100 and 200 nM). Cells were labeled with anti-acetylated α-tubulin (green). Nuclei were labeled with ToPro-3 (blue). HT and LT cultures exhibited a reduction in the number of ciliated cells (e and i, respectively). ATRA increased the number of ciliated cells in LT (f, g and h) and HT (j, k and l), being more notable in HT. In control, ATRA decreased the number of ciliated cells. Panel k shows the zoom of a cell with two cilia. (B to E) Quantification of number of ciliated cells. Mean ± SEM of three individual experiments from three different patients are shown. (**B**) *P<0.*05* versus control; ***P<0.001 versus control. (**C**) **P<0.01 and **P<0.01 versus control with ATRA 0 nM. (**D**) **P<0.01 and *P<0.05 versus LT with ATRA 0 nM. (**E**) *P<0.05 and **P<0.01 versus HT with ATRA 0 nM. ATRA, all trans retinoic acid; LT, low transporter; HT, high transporter.

Cilia length among groups did not show differences (Figure 7A, panels a, e and i, and Figure 7B). In control (Figure 7A, panels c and d) and HT cultures (Figure 7A, panels j, k and l), ATRA increased cilia length. In HT, this effect was observed at 50 nM (Figure 7E), whereas in control it was present at 100 nM (Figure 7C). In LT, ATRA did not have effect. Ciliated cells in both LT (Figure 8A, panel e) and HT (Figure 8A, panel i) were less abundant compared to control (Figure 8A, panels a, and Figure 8B). ATRA increased percentage of ciliated cells in LT and HT, being more evident in HT cultures (Figure 8E). In control cultures, ATRA (100 and 200 nM) decreased percentage of ciliated cells (Figure 8C). 

### Microvilli number and size was decreased in HPMCs from HT

**Figure 9:**
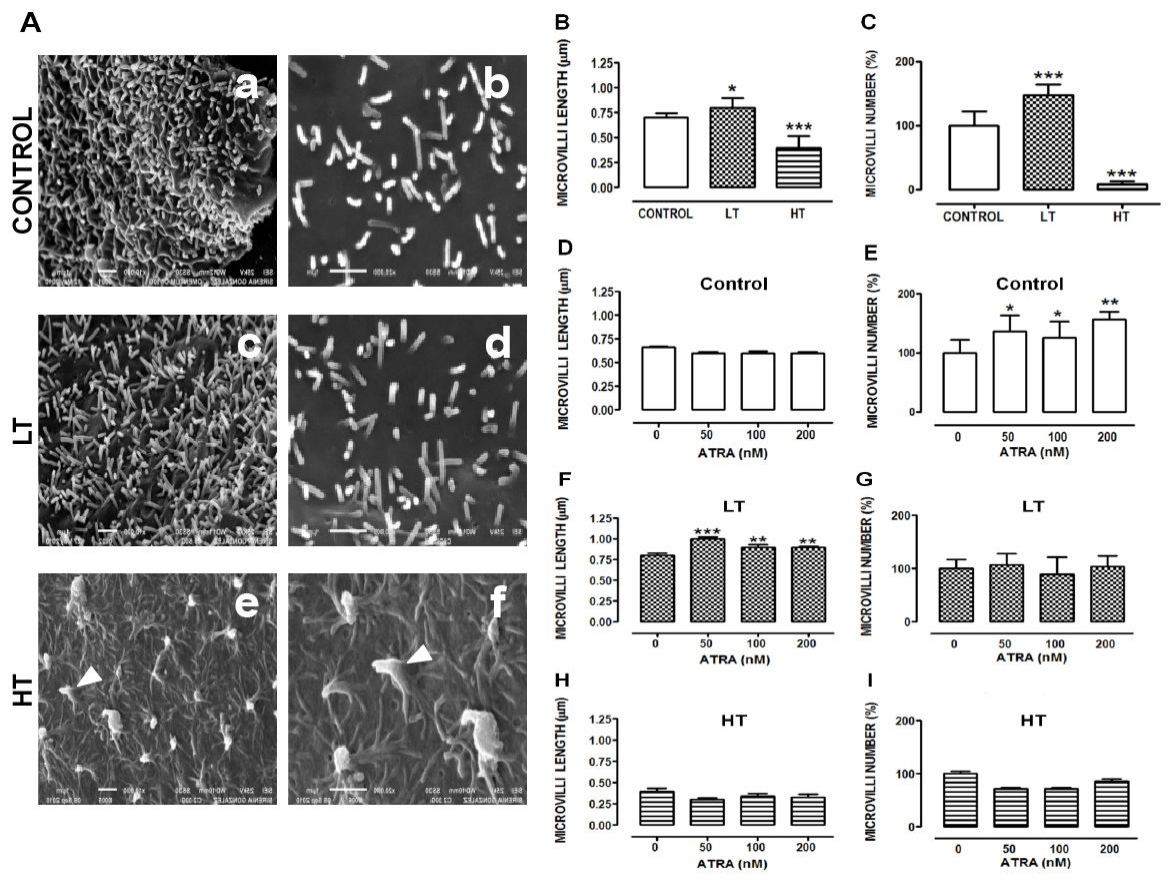
Microvilli number and size were reduced in cells from HT. (**A**) Omentum derived-mesothelial cells (control) and effluent-derived mesothelial cells from LT and HT grown until confluence in the presence of ATRA (0, 50, 100 and 200 nM). In HT, microvilli were scarce (e) and exhibited a truncated morphology (f, arrowhead). HT cell surface showed a rough appearance, while in control (b) and LT (d) it was smooth. Measurement of length (**B**, **D**, **F** and **H**) and quantification of number of microvilli (**C**, **E**, **G** and **I**). Microvilli were shorter and almost absent in HT. In contrast, they were longer and abundant in LT (**B** and **C**). ATRA increased microvilli length in LT (**F**), but it did not have effect on HT (**H**). Scanning electron microscopy, x20000, bar=1µm. Mean ± SEM of three separate experiments from three different patients are shown. (**B**) *P<0.05 and***P<0.001 versus control. (**C**) ***P<0.001 versus control. (**E**) *P<0.05 and **P<0.01 versus control with ATRA 0 nM. (**F**) ***P<0.001 and **P<0.01 versus LT with ATRA 0 nM. ATRA, all trans retinoic acid; LT, low transporter; HT, high transporter.

Control and LT cells exhibited numerous microvilli on their surface (Figure 9A, panels a and c, respectively), whereas in HT they were notably reduced (Figure 9A, panel e). In control and LT, microvilli were observed as single cylindrical structures (Figure 9A, panels b and d, respectively). In contrast, HT microvilli were fused forming protrusions (Figure 9A, panels e and f, arrowheads). Membranal surface in HT showed rough appearance (Figure 9A, panel f), while in control and LT it was smooth (Figure 9A, panels b and d). LT microvilli were larger and more abundant (48%) than in control, while in HT, they were shorter, with an important reduction (91%) in their number (Figure 9, B and C). ATRA increased microvilli number in control cells (Figure 9E and Figure S2) and the microvilli length in LT (Figure 9F and Figure S3). 

## Discussion

Mesothelium acts as permeability barrier to regulate passage of water and solutes, between the intravascular compartment and the peritoneal cavity. Effectiveness to remove water and solutes varies between patients. We studied the morphological characteristics of human peritoneal mesothelial cells (HPMCs) obtained from peritoneal effluents of low transporters (LT) and high transporters (HT) patients and compared them with non-uremic HPMCs (control). 

HPMCs cultures from dialysate effluents display several morphologies: cobblestone-like, transitional, fibroblastic-like cells or mixed populations [10]. Occasional hypertrophic cells are observed mixed with HPMCs of normal size [13]. We show, for the first time to our knowledge, that HPMCS from patients undergoing continuous ambulatory peritoneal dialysis (CAPD) and classified as LT or HT exhibited clear differences in morphology, in spite of similar time in dialysis (Table 1). LT showed augmented average cell size compared with control, and hypertrophic cells. In HT, hypertrophic cells mixed with cells of normal size were observed. It must be emphasized that these alterations are similar to those observed in senescent cells. They are characterized by growth arrest in G_1_ phase of the cell cycle, hypertrophic morphology, increased expression of cell cycle inhibitor genes, and senescence markers [11]. One distinctive feature of aged cells is hypertrophy. Cellular hypertrophy has also been associated with hyperglycemia as observed after exposure of renal mesangial cells to elevated glucose [26]. Similarly HPMCs exposed to high glucose displayed hypertrophy [27]. Our cultures were obtained from CAPD patients exposed to dialysis solutions containing high glucose concentration. Therefore presence of senescent phenotype in our cultures is in agreement with previous reports. 

During epithelial to mesenchymal transition (EMT) polarized cells are converted into fibroblast-like cells, capable of locomotion [12]. These cells lack of transport mechanisms present in mesothelial cells and they are not suitable for adequate dialysis. We studied expression of epithelial (cytokeratin, claudin-1, occludin and ZO-1) and mesenchymal (vimentin and α-SMA) markers in cultured cells, to assess the presence of EMT. LT and HT cells exhibited cytokeratin and vimentin cytoskeletal disorganization and stress fiber formation, disclosed by α-SMA staining. Vimentin and α-SMA expression were notably increased in LT cultures. In contrast, the expression of epithelial markers (claudin-1, occludin and ZO-1) was significant reduced in LT and also in HT. LT and HT cells were positive for both epithelial and mesenchymal markers. This colocalization defines an intermediate phenotype of EMT [12], indicating that HPMCs from these patients displayed some markers of EMT, but preserved their mesothelial phenotype. 

Transforming growth factor-β1 (TGF-β1) is a key mediator of EMT in cultured HPMCs [17]. Ksiazek et al. (2007) reported development of senescent phenotype, mediated by TGF-β1, in HPMCs exposed to high glucose [27]. This factor was increased in LT and HT cultures. Our data suggest that senescent phenotype and the presence of EMT markers in LT and HT cultures might be related to TGF-β1 overexpression. It is noteworthy that overexpression of this factor showed a different degree in HT and LT (higher in LT), in spite of a similar time in dialysis.

Microvilli and cilia act as receivers and transducers of information from environmental stimuli [28], mediated by the presence of receptors, transporters and channels in ciliary and microvilli membranes [29,30]. They protect mesothelial surface from frictional injury by entrapment of water and serous exudates and by regulation of surfactants secretion. Therefore, reduction in the number of these structures may have deleterious consequences on peritoneal function. HPMCs cultures from control and patients in CAPD exhibited multi-ciliated cells, with two or more cilia protruding from the same or adjacent ciliary tuft. Cilium average length in control group was 4.1±0.2µm, similar to that reported by Bird et al (2004) in HPMCs [24]. Cilium length was not altered in LT and HT. LT and HT cultures showed a reduced number of ciliated cells compared to control, being more evident in HT. After peritoneal dialysis microvilli suffer shortening and gradual reduction in their number until its total disappearance (after 15 months in dialysis) [9]. HT showed a marked reduction in the size and in the number of microvilli (91%) similar to that described by Fang et al [9]. These authors, however, did not specify the transport characteristics (HT or LT) of the patients. In contrast, LT exhibited abundant long microvilli. Our cultures were obtained from patients with more than 15 months in CAPD (Table 1). Classification of patients as LT or HT was performed at the initiation of CAPD. Therefore differences that we describe do not seem to be related to dialysis deleterious effects associated to duration or composition of dialysis solutions. The density of microvilli on regenerating mesothelial cells may vary and is dependent on the anionic charge of the glycocalyx [31]. Kultti et al. (2006) reported that presence of hyaluronan in the glycocalyx of MCF7 cells is essential for the formation of microvilli and that the length of these protrusions is dependent on hyaluronan synthases (HAS) activity and the rate of hyaluronan synthesis [32]. Synthesis of microvilli on mesothelial cells has not been fully investigated, but is possible that is regulated by hyaluronan as observed in MCF7 cells. HAS are plasma membrane enzymes. HT showed membrane alterations. Absence of microvilli in HT might be associated with alterations in their HAS activity. Microvilli formation occurs during differentiation or growth arrest [30,33]. It occurs in G_0_/G_1_ predifferentiation state and after differentiation onset. LT cultures exhibited senescent cells characterized by growth arrest in G_1_ phase which might be associated with the abundant microvilli observed in these cultures.

All trans retinoic acid (ATRA) regulates critical genetic programs controlling development and homeostasis, cell proliferation and differentiation, as well as death and survival [34]. ATRA regulates gene expression through nuclear receptors, the retinoic acid receptor (RAR) which is composed of three subtypes (α, β and γ) [35]. In mesothelial cells retinoic acid plays an important regulating role in cell differentiation [20]. We describe, for the first time to our knowledge, that ATRA elicited a differential effect in control, LT and HT peritoneal mesothelial cells. In LT, ATRA reverted hypertrophy, reorganized cytokeratin and vimentin networks, reduced vimentin and α-SMA expression, and increased ZO-1 expression. In HT, ATRA improved cytokeratin and vimentin organization, decreased vimentin and α-SMA expression and augmented claudin-1, occludin and ZO-1 expression, but it did not have effect on cell morphology. 

TGF-β1 is increased in senescent HPMCs and it induces epithelial-mesenchymal transition (EMT). Depending on the cellular type, ATRA may increase or decrease TGF-β1 expression [36–39]. In a rat model of peritoneal dialysis, ATRA decreased peritoneal TGF-β1 expression [40]. In agreement with this finding, we showed that ATRA decreased TGF-β1 expression in human cells. This effect was observed only in HPMCs from LT. Senescent phenotype in LT might be associated with the high expression of TGF-β1 in these cultures, since the decrease in TGF-β1 expression induced by ATRA was accompanied by decrease in the number of hypertrophic cells in these cultures. p38 mitogen-activated protein kinase (p38MAPK) inhibits EMT induction in HPMCs [14]. Retinoic acid (RA) activates p38MAPK [41]. This activation requires the formation of a complex between RARα, present in membrane lipid rafts, with Gαq in response to RA [41]. ATRA reversed the tendency to EMT in LT and HT, being more evident in LT. ATRA effect might be through a possible involvement of p38 pathway. Compared with LT, HT exhibited cell surface alterations, which might explain that HT cells were less sensitive to ATRA treatment than LT cells.

RA and activation of RAR induces ciliogenesis in maxillary and paranasal sinus mucosa [42–45]. We showed that ATRA increased the number and length of cilia in HPMCs from CAPD patients, being more evident in HT. Mechanisms of ciliogenesis associated to RA are poorly understood. Hill et al. (1998) [46] reported that RA induces upregulation of 15 lipoxygenase and prostaglandin-H synthase, enzymes associated with ciliogenesis, in human tracheobronchial epithelial cells. It is possible that ATRA might induce ciliogenesis in LT and HT by this mechanism. In control group a diminution in ciliated cells was observed at high ATRA concentrations. Cilia are post mitotic structures present in cells in G0/G1 and beginning of S phase, when cells are not in a proliferative state [28]. Mesothelial cells with a flattened appearance are characteristic of a proliferative phase [47]. In control, ATRA (200 nM) increased the number of cells with flattened appearance. This might explain ciliated cells reduction by ATRA in these cultures. Effect of ATRA on microvilli was also explored. It increased microvilli number in control, and increased their length in LT. Hyaluronan induces microvilli formation. RAR are involved in HAS mRNA synthesis [48]. Sanna et al. (2008) showed that ATRA increased synthesis of hyaluronan and expression of HAS in rat keratinocytes cultures [49]. It is probable that ATRA had a similar effect in control and LT HPMCs. 

Injury in mesothelial cells results in structural changes in the peritoneal membrane. These structural changes are associated with a loss of ultrafiltration and solute clearance that leads to technique failure and unfavorable clinical outcome. Transport of solutes in LT is slow, while in HT it is fast. Microvilli increase solute transport surface area. However, Lange et al. reported that the actin microvillar filaments may act as an effective diffusion barrier [30,50]. LT possess abundant microvilli, but in HT they were almost absent. According to Lange et al., the slow transport in LT might be due to a diffusion barrier formed by their abundant and long microvilli, while fast transport in HT might be related to their absence. Molecular movement through mesothelium is governed by their ionic charge. Most mammalian cells have a net negative surface charge, 90 per cent of the total cell surface charged groups are anionic [31]. Anionic sites are localized within mesothelial glycocalyx, submesothelium, and along the basement membrane. Reduction in anionic sites along the submesothelial basement membrane results in increased transperitoneal passage of proteins [51]. If cell surface morphology is altered, there is a change in their surface charge. HT showed severe membrane alterations that might change membrane charge and results in abnormal mesothelial transport. This might be associated with the fast transport observed in these patients. 

Deterioration of peritoneal function is a severe complication for the survival of patients undergoing dialysis. EMT during dialysis plays an important role in the development of peritoneal fibrosis and failure of ultrafiltration [52]. We showed that HPMCs cultures from LT and HT patients exhibit differential alterations in EMT markers distribution and expression, and also in TGF- β1expression (inducer of EMT in HPMCs). The most dramatic change was observed in HT cells. They undergo EMT, showed a pronounced reduction in microvilli, and severe cilia and membrane alterations. In LT patients, long-term exposure to mechanical denudation and TGF-β1 overexpression may induce a complete transition of the mesothelial cells to fibroblastic phenotype and accelerate failure of ultrafiltration. We suggest that the beneficial effects of ATRA that we describe might delay the alterations observed in LT and HT, and prolong the time that dialysis fulfill its therapeutic purpose.

## Conclusion

HPMCs from LT and HT in CAPD exhibited morphological and structural alterations that might be associated with differences in the transport of solutes and water. ATRA might be a therapeutic alternative to maintain the mesothelial integrity in CAPD patients, since this retinoid improved morphology, reverted EMT tendency, decreased TGF-β1 (profibrotic factor) and induced ciliogenesis. All these effects are in favor of prolonging an adequate peritoneal function, with increased life expectancy. Therefore ATRA might have a beneficial effect on the peritoneal function and transport.

## Supporting Information

Figure S1 **HT cilia exhibited diverse morphologies.** One (a and b), two (c) or three (d and e) cilia were observed in HT cells. Cilia showed thinning from the base to the tip (a and b, arrows). In multi-ciliated cells, cilia protruding from the same ciliary tuft (c, d and e, arrowhead) and seem to be fused at their tips. Cilia showed an irregular pattern, with a “pearl necklace” (c and d, empty arrowhead) or “branched” (f, empty arrowhead) appearance. Frequently, the ciliary tip was laying on the cell surface (g and h, white arrows). Scanning electron microscopy, x10000, bar=1µm. HT, high transporter.
(TIF)

Figure S2 **ATRA increased the number of microvilli in control HPMCs.** Scanning electron microscopy of control HPMCs treated with ATRA 0, 50, 100 and 200 nM until confluence. x10000, bar=1µm. ATRA, all trans retinoic acid; HPMCs, human peritoneal mesothelial cells.
(TIF)

Figure S3 **ATRA increased microvilli length in LT HPMCs.** Scanning electron microscopy of LT HPMCs treated with ATRA 0, 50, 100 and 200 nM until confluence. x10000, bar=1µm. ATRA, all trans retinoic acid; HPMCs, human peritoneal mesothelial cells; LT, low transporter.
(TIF)
