# Supplementary figures and images for: Retinoic Acid Improves Morphology of Cultured Peritoneal Mesothelial Cells from Patients Undergoing Dialysis
Source: PLoS One. 2013 Nov 4;8(11):e79678. doi: 10.1371/journal.pone.0079678 (PMC3817121; doi:10.1371/journal.pone.0079678)

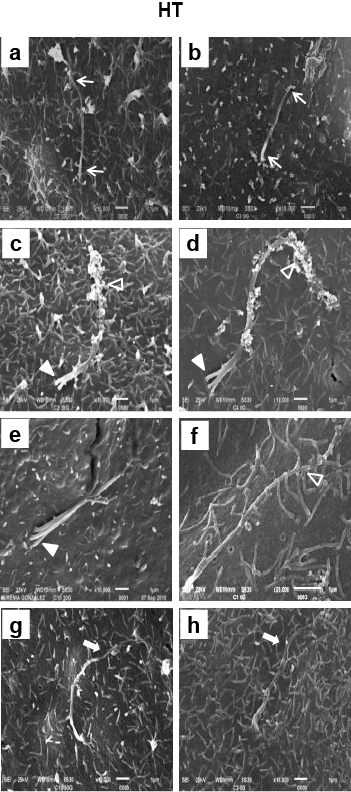

Supplement: Figure S1 — HT cilia exhibited diverse morphologies. One (a and b), two (c) or three (d and e) cilia were observed in HT cells. Cilia showed thinning from the base to the tip (a and b, arrows). In multi-ciliated cells, cilia protruding from the same ciliary tuft (c, d and e, arrowhead) and seem to be fused at their tips. Cilia showed an irregular pattern, with a “pearl necklace” (c and d, empty arrowhead) or “branched” (f, empty arrowhead) appearance. Frequently, the ciliary tip was laying on the cell surface (g and h, white arrows). Scanning electron microscopy, x10000, bar=1µm. HT, high transporter. (TIF) [file pone.0079678.s001.tif]

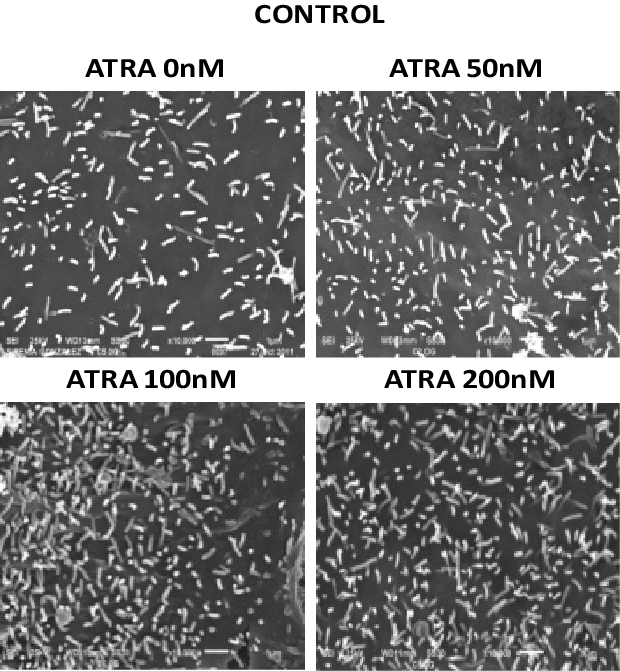

Supplement: Figure S2 — ATRA increased the number of microvilli in control HPMCs. Scanning electron microscopy of control HPMCs treated with ATRA 0, 50, 100 and 200 nM until confluence. x10000, bar=1µm. ATRA, all trans retinoic acid; HPMCs, human peritoneal mesothelial cells. (TIF) [file pone.0079678.s002.tif]

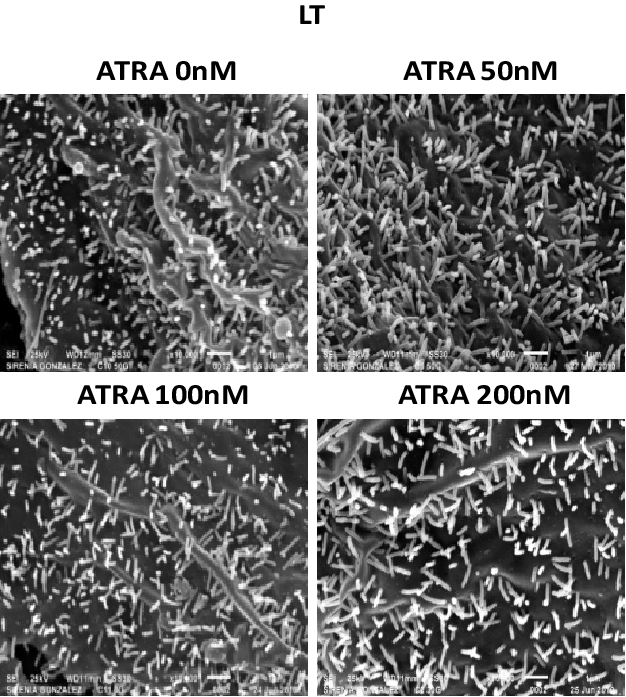

Supplement: Figure S3 — ATRA increased microvilli length in LT HPMCs. Scanning electron microscopy of LT HPMCs treated with ATRA 0, 50, 100 and 200 nM until confluence. x10000, bar=1µm. ATRA, all trans retinoic acid; HPMCs, human peritoneal mesothelial cells; LT, low transporter. (TIF) [file pone.0079678.s003.tif]
